# Real-time multicenter semantic segmentation for laparoscopic gastrectomy: development, external validation and operating room deployment of an AI navigation system

**DOI:** 10.1016/j.mmr.2026.100040

**Published:** 2026-06-03

**Authors:** Ke-Cheng Zhang, Di Wu, Can-Rong Lu, Zhi Qiao, Wen-Tong Xu, Feng-Lin Liu, Jing-Yu Deng, Li Yang, Lei Guo, Tian-Yu Xie

**Affiliations:** a Department of General Surgery, First Medical Centre, Chinese PLA General Hospital, Beijing 100853, China; b Department of General Surgery, Sixth Medical Centre, Chinese PLA General Hospital, Beijing 100048, China; c Department of Gastric Surgery, Fudan University Affiliated Cancer Hospital, Shanghai 200032, China; d Department of Gastric Surgery, Tianjin Medical University Cancer Institute & Hospital, Tianjin 300060, China; e Department of Gastrointestinal Surgery, Liyang Branch of Jiangsu Province Hospital, Jiangsu 213300, China

**Keywords:** Artificial intelligence (AI), Computer vision, Semantic segmentation, Laparoscopic gastrectomy, Intraoperative navigation, Multicenter study, Real-time inference

## Abstract

**Background:**

Laparoscopic gastrectomy is technically demanding and errors often arise from limited situational awareness. Artificial intelligence (AI)-based computer vision may enhance intraoperative recognition of instruments and anatomy, but evidence for generalizable, real-time deployment remains limited.

**Methods:**

We conducted a multicenter study using laparoscopic gastrectomy videos from 4 tertiary hospitals. Data were split by institution into training, internal test, and external validation sets. A You Only Look Once version 10 (YOLOv10)-based model was developed to recognize 18 categories (11 instruments and 7 organs) and integrated into a lightweight user interface for bedside use. Mean average precision at Intersection over Union (IoU) 0.50 (mAP@50) and AP@[0.50–0.95] for boxes and masks, recall, and F1 score were calculated. The feasibility of live operating-room deployment was assessed. Surgeon cognitive workload was assessed using the National Aeronautics and Space Administration Task Load Index (NASA-TLX), and system usability was evaluated with the System Usability Scale (SUS).

**Results:**

The training set comprised 4308 frames (41,066 labels); the internal testing set, 1803 frames (17,211 labels); and the external validation set, 721 frames (6685 labels). Training performance reached box precision 0.896, recall 0.891, mAP@50 0.937, and AP@[0.50–0.95] 0.846; mask AP@[0.50–0.95] was 0.809. On the internal testing set, box AP@[0.50–0.95] was 0.670 and mask AP@[0.50–0.95] 0.642. External validation achieved box precision 0.798, recall 0.773, F1 score 0.785, mAP@50 0.832, and AP@[0.50–0.95] 0.684; mask mAP@50 was 0.836 and AP@[0.50–0.95] 0.658. High-performing classes included stapler, ultrasonic scalpel, and forceps, whereas omentum and pancreas were relatively lower. The system operated in real time during laparoscopic gastrectomy on a laptop-grade GPU, providing on-demand overlays without workflow disruption. The NASA-TLX score was 26.72±6.51, indicating low cognitive burden, and the SUS score was 78.33±5.20, demonstrating good usability.

**Conclusions:**

We present development, external validation, and live deployment of a real-time AI navigation system for laparoscopic gastrectomy across multiple centers. The model demonstrated robust detection and segmentation of instruments and organs and was feasible for bedside use. Prospective trials are warranted to evaluate effects on intraoperative safety, efficiency, and training.

## Background

Gastric cancer remains a major global health burden and is the fifth leading cause of cancer-related morbidity and mortality [1]. GLOBOCAN 2022 estimates nearly 1 million new cases and more than 650,000 deaths annually [1], [2]. Incidence varies markedly by geography, with a 15- to 20-fold difference between high- and low-incidence regions [3]. The highest rates occur in Asia, South America, and Eastern Europe, whereas Western Europe, Australia, and North America report the lowest [4]. China alone contributes almost 50% of newly diagnosed non-cardia gastric cancers [5] with an incidence of 25.41 per 100,000 people reported in 2022 [6]. Although long-term trends suggest declining incidence and mortality [7], early-onset disease is increasing, particularly among individuals born between 1980 and 1994, whose incidence is approximately double that of cohorts born in the 1950s [8].

Surgical resection is the cornerstone of curative treatment, and laparoscopic approaches now predominate. In China, a nationwide cohort from 53 leading hospitals across 27 provinces reported that among 90,292 surgically treated patients, the proportion undergoing laparoscopy exceeded that of laparotomy (49.51% vs. 47.67%) [9]. In Korea, the proportion of laparoscopic gastrectomy rose from 6.6% in 2004 to 70.8% in 2023 [10]. Japan has seen a steady 3%–5% annual increase over the past decade [11]. However, laparoscopic gastrectomy is technically demanding, with a relatively long learning curve [12]. The Dutch Upper Gastrointestinal Cancer Audit reported postoperative complications as high as 28.5% after laparoscopic gastrectomy [13].

Nearly half (48%) of preventable in-hospital adverse events arise intraoperatively [14]. Visual navigation systems that provide real-time spatial relationships between instruments and anatomy, a Global Positioning System (GPS) for the body, can enhance precision, safety, and efficiency [15]. Unlike solid-organ procedures that can leverage preoperative computed tomography-based models, operations on hollow organs such as the stomach are challenged by dynamic deformation due to traction and insufflation, limiting the utility of static preoperative models. Consequently, given the complex vascular and visceral anatomy surrounding the stomach, real-time, intraoperative recognition is essential for laparoscopic gastrectomy.

Minimally invasive procedures inherently benefit from improved visualization and precision. As surgical fields continue to evolve, artificial intelligence (AI) has emerged as a vital technology to bridge the gap between human perceptual limitations and the demands of modern surgical practice. AI has transformed diagnostic imaging, prognostication, and perioperative risk stratification [16]. Yet, intraoperative AI for real-time guidance remains underdeveloped for gastrectomy because of the complexity of the surgical scene, including patient-specific anatomy, camera motion, smoke, and variable image quality. Using multicenter data, we developed an AI model for real-time semantic segmentation of instruments and organs during laparoscopic gastrectomy and assessed its performance, including live intraoperative deployment.

## Methods

### Ethics approval

The study was centrally reviewed and approved by the Ethics Committee of the leading center, Chinese PLA General Hospital and shared among multi-centers (S2024-076-01). Written informed consent was obtained from participants. Each participating center (Fudan University Affiliated Cancer Hospital, Liyang Branch of Jiangsu Province Hospital, and Tianjin Medical University Cancer Institute & Hospital) filed the necessary regulatory records in accordance with their respective institutional requirements. All video data collection, de-identification, storage, and inter-institutional sharing procedures adhered strictly to institutional data governance policies and the ethical principles outlined in the Declaration of Helsinki.

### Video dataset

Laparoscopic gastrectomy videos from 4 tertiary hospitals (Chinese PLA General Hospital, Fudan University Affiliated Cancer Hospital, Liyang Branch of Jiangsu Province Hospital, and Tianjin Medical University Cancer Institute & Hospital) were collected. Surgeons routinely recorded operations for review, education, and quality improvement, with recording performed at the surgeon’s discretion. The inclusion criteria were totally laparoscopic gastrectomy performed between May 2022 and December 2024, and high-quality video with 1920×1080 resolution at 25 frames per second. Videos were converted to Joint Photographic Experts Group (JPEG) format images at 5-second intervals. The 5-second sampling interval was selected after systematic empirical evaluation. Initial experiments tested 1-second, 2-second, 5-second, and 10-second intervals on a pilot dataset. The 1-second interval yielded approximately 90% redundant frames with minimal anatomical variation between consecutive frames, creating substantial annotation burden without meaningfully improving model diversity. By contrast, the 10-second interval missed critical surgical transitions and rare events such as Hem-o-lok application for vascular ligation, reducing the representation of these already-limited categories in the training data. The 5-second interval provided an optimal balance, ensuring frame diversity and capturing the full range of surgical scenarios while avoiding omission of important events. Visually redundant and blurry frames were removed to ensure clarity.

Technical specifications by institution: Chinese PLA General Hospital employed the Aesculap EinsteinVision 2.0 Endoscopy System with an EinsteinVision 2.0 camera and 300 W Xenon light source or the Karl Storz IMAGE1 S 3D system with 300 W Xenon light source (IMAGE1 S enhancement modes disabled); Fudan University Affiliated Cancer Hospital, Liyang Branch of Jiangsu Province Hospital, and Tianjin Medical University Cancer Institute & Hospital all used Karl Storz IMAGE1 S 3D systems with 300 W Xenon light sources (IMAGE1 S enhancement disabled). Dedicated smoke evacuation devices are not routinely used for laparoscopic gastrectomy in China; surgical aspirators were used for intermittent smoke evacuation. All videos were captured in standard white-light mode without proprietary image enhancement algorithms to maximize generalizability across imaging platforms.

### Frames annotation

Two board-certified surgeons (each with experience in at least 50 gastric cancer operations) independently annotated frames using Labelme. Eighteen categories were annotated by semantic segmentation in each frame (Additional file 1**:** Fig. S1): needle, needle holder, ultrasonic scalpel, aspirator, colon, forceps, gallbladder, gauze, Hem-o-lok, Hem-o-lok forceps, hook, intestine, liver, omentum, pancreas, stapler, stomach, and trocar. Disagreements were resolved through consensus discussion. The annotated frames are provided in the Additional file 2 (https://figshare.com/s/0c0210ede8a309f83182).

### Model development and evaluation metrics

We trained, validated, and tested an AI model based on You Only Look Once version 10 (YOLOv10), using the default architecture and finetuning on our dataset [17]. Training used standard data augmentation and early stopping. Loss and performance curves were monitored to assess convergence over 100 training epochs. We assessed performance using: 1) Intersection over Union (IoU) [18]: IoU=Area of Intersection/Area of Union between predicted and ground-truth box or mask; 2) Precision: proportion of true positives among all positive predictions; 3) Recall: proportion of true positives among all ground-truth positives; 4) Average precision (AP): area under the precision-recall curve at a given IoU threshold; AP@50 denotes AP at IoU=0.50; AP@[0.50–0.95] denotes averaged AP across IoU thresholds from 0.50 to 0.95 in steps of 0.05; 5) Mean average precision (mAP): mean AP across classes; mAP@50 averages AP@50 over all classes; 6) F1 score: 2×(precision × recall)/(precision + recall).

### Intraoperative deployment

The constructed AI model was integrated into a user interface (UI) [Additional file 3 (https://figshare.com/s/2cafd49139b6e6e02b41)]. During surgery, we used either the Aesculap EinsteinVision 3D HD laparoscopic system (B. Braun, Germany) or the Karl Storz 3D IMAGE1 S system (Karl Storz, Germany). The serial digital interface connected to a Jiuyinjiushi HD90 recording unit to capture the video stream, which was then fed into a laptop for real-time inference (Intel Core i9, up to 5.4 GHz; 64 GB DDR5 RAM; NVIDIA GeForce RTX 4070, 8 GB). The UI overlaid color-coded contours of selected instruments and organs in real time, with optional toggling of classes to reduce visual clutter. An external monitor was connected to the laptop to provide a clearer display of real-time AI segmentation results. Gastrectomy and lymph node dissection were performed according to the Japanese Gastric Cancer Treatment Guidelines [19]. Digestive reconstruction was at the surgeon’s discretion.

### Cognitive load and system usability

Surgeon’s cognitive workload was evaluated by the National Aeronautics and Space Administration Task Load Index (NASA-TLX). NASA-TLX is a widely used questionnaire designed to measure the cognitive load during task performance. It consists of 6 domains: mental demand, physical demand, temporal demand, performance, effort, and frustration. Each domain is rated from 0 to 100, and the unweighted overall workload is the average of the 6 domain scores. NASA-TLX is the most commonly used tool for assessing cognitive workload and has been extensively explored in surgical research [20], [21].

The perceived usability of the AI‑based navigation system was evaluated using the System Usability Scale (SUS). The SUS is a validated instrument of 10 items assessing overall usability of interactive systems [22], [23]. Each item is scored on a 5‑point Likert scale from 1 (“strongly disagree”) to 5 (“strongly agree”). Following standard scoring procedures, item responses were converted to a composite SUS score ranging from 0 (poor usability) to 100 (excellent usability), with higher scores indicating better perceived usability. For odd‑numbered items, the score contribution was (response−1), and for even‑numbered items was (5−response). The sum of these contributions was multiplied by 2.5 to obtain the total SUS score (0–100). The chief surgeon completed the NASA-TLX and SUS assessments after each procedure (Additional file 1**: Questionnaire**).

### Statistical analysis

Normally distributed continuous variables were summarized as mean ± standard deviation (SD), whereas variables departing from normality were reported as median with interquartile range. Categorical data were expressed as counts. Model classification behavior was inspected through confusion matrices in both raw-count and column-normalized forms. To gauge generalizability, performance metrics were contrasted among the training, internal testing, and external validation sets, with absolute changes in AP@[0.50–0.95] used to quantify the degree of variation across datasets and institutions. All analyses were carried out using Python (version 3.10) and SPSS (version 25.0), and model training together with metric computation was performed within the Ultralytics YOLOv10 framework.

## Results

### Dataset

**Fig. 1:**
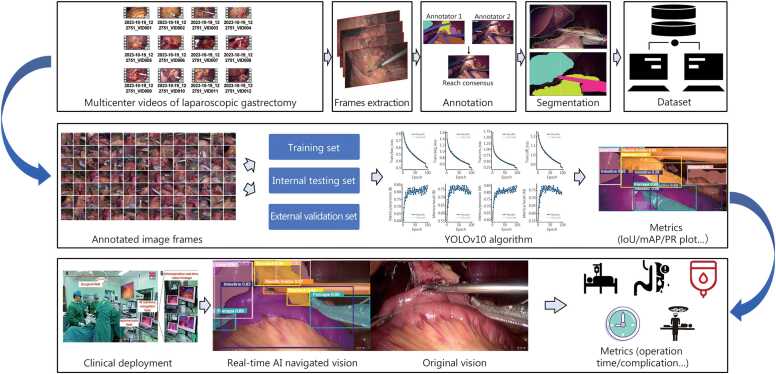
Study flowchart. The pipeline proceeds from multicenter video acquisition, through meticulous annotation and YOLOv10 training, internal testing and external validation, to real-time intraoperative deployment and clinical outcome assessment. YOLOv10. You Only Look Once version 10; IoU. Intersection over Union; mAP. Mean average precision; PR plot. Precision-recall plot; AI. Artificial intelligence.

The study design is summarized in Fig. 1. We collected 18 videos from 4 tertiary hospitals. Of these, 15 were from the Chinese PLA General Hospital and were split into a training set (10 videos) and an internal testing set (5 videos). Three additional external videos (one per external hospital) formed the external validation set. Each frame was annotated by semantic segmentation. After removing redundant and blurry frames, the training, internal test, and external validation sets comprised 4308, 1803, and 721 frames, respectively, with 41,066, 17,211, and 6685 total labels across the 18 categories. No image was repeated across the three independent datasets.

### Training performance

**Fig. 2:**
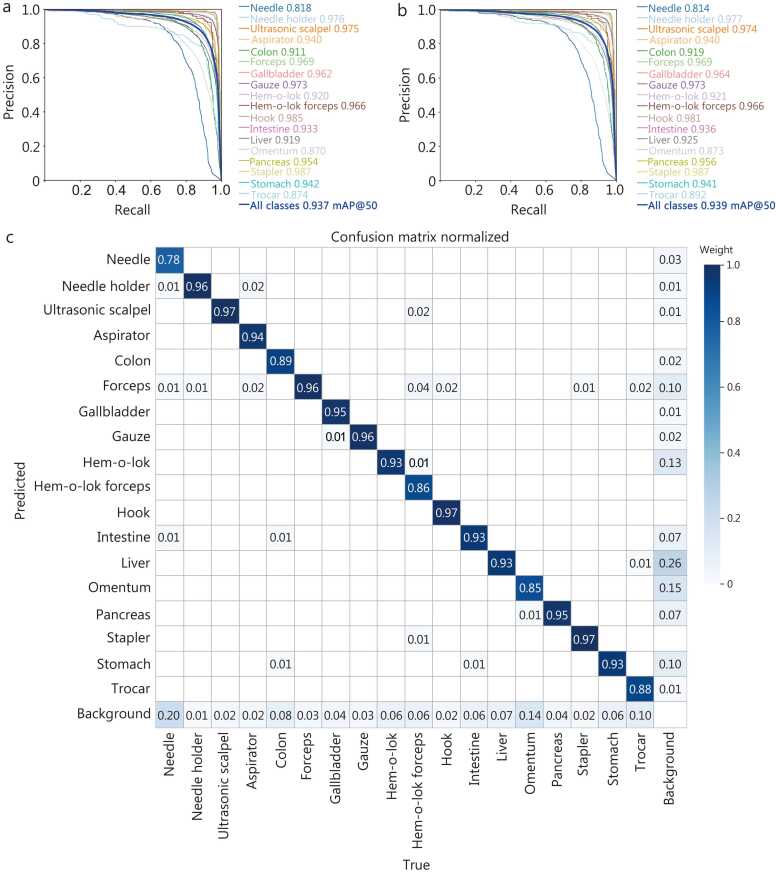
Training set: precision-recall (PR) plot for box bounding (**a**) and mask segmentation (**b**), and normalized confusion matrix heatmaps (**c**). For the PR plot, the X-axis (“recall”) ranges from 0 to 1.0 and represents the proportion of actual positive instances (e.g., objects in the image) that the model correctly detects; higher recall indicates fewer missed objects. The Y-axis (“precision”) ranges from 0 to 1.0 and represents the proportion of the model’s positive predictions that are correct (i.e., fewer false positives). PR plots illustrate the trade-off between precision and recall across different detection thresholds. Each subplot contains 18 distinct curves, each shown in a different color (e.g., blue, orange, green, red, purple), representing the individual object classes. At the bottom of the legend, the overall mean average precision (mAP) for all classes is displayed. For the normalized confusion matrix heatmaps, rows represent the predicted labels and columns represent the true (ground-truth) labels. Each cell’s color and value indicate the proportion of true instances that were predicted as a particular class. Values range from 0 to 1.0 and sum to 1.0 within each column. Cells are shaded along a blue gradient-light blue for low values (approximately 0) and dark blue for high values (approximately 1.0). Text in each cell displays the exact normalized value. Diagonal cells represent correctly classified instances (e.g., a diagonal value of 0.97 means that 97% of stapler instances were correctly predicted). Off-diagonal cells represent misclassifications. For example, the cell at row “Forceps” and column “Hem-o-lok forceps” has a value of 0.04, indicating that 4% of all true Hem-o-lok forceps instances were incorrectly predicted as forceps.

After 50 epochs, the loss curves plateaued and the performance metrics stabilized, indicating that the model was well-fitted (Additional file 1**:** Fig. S2). The training set performance is summarized in Table 1. For all classes combined, box bounding detection achieved precision 0.896, recall 0.891, mAP@50 0.937 (Fig. 2**a**), AP@[0.50–0.95] 0.846, and F1 score 0.893. Semantic mask segmentation demonstrated similar but slightly lower performance [precision 0.898, recall 0.893, mAP@50 0.939 (Fig. 2**b**), AP@[0.50–0.95] 0.809, and F1 score 0.895].

**Table 1:** Training set.

Category	Images	Label count	Box bounding metrics				Mask segmentation metrics			
			Precision	Recall	AP@[0.50–0.95]	F1 score	Precision	Recall	AP@[0.50–0.95]	F1 score
All	4308	41,066	0.896	0.891	0.846	0.893	0.898	0.893	0.809	0.895
Needle	642	976	0.867	0.696	0.644	0.772	0.870	0.698	0.499	0.775
Needle holder	882	964	0.956	0.962	0.927	0.959	0.956	0.962	0.885	0.959
Ultrasonic scalpel	1380	1452	0.970	0.969	0.949	0.970	0.970	0.969	0.923	0.970
Aspirator	216	224	0.812	0.960	0.891	0.879	0.812	0.960	0.871	0.879
Colon	631	852	0.864	0.840	0.763	0.852	0.867	0.844	0.739	0.855
Forceps	3744	6864	0.924	0.955	0.909	0.939	0.925	0.956	0.847	0.940
Gallbladder	667	735	0.905	0.932	0.898	0.918	0.905	0.932	0.874	0.918
Gauze	1367	1692	0.927	0.953	0.903	0.940	0.927	0.952	0.876	0.939
Hem-o-lok	2051	4045	0.844	0.887	0.782	0.865	0.845	0.888	0.688	0.866
Hem-o-lok forceps	120	140	0.946	0.880	0.895	0.912	0.946	0.880	0.867	0.912
Hook	321	327	0.965	0.972	0.947	0.968	0.962	0.969	0.921	0.965
Intestine	1661	2958	0.881	0.892	0.822	0.887	0.884	0.895	0.800	0.889
Liver	3461	7294	0.843	0.859	0.801	0.851	0.853	0.869	0.778	0.861
Omentum	2390	4440	0.848	0.748	0.702	0.795	0.853	0.752	0.660	0.799
Pancreas	2093	2886	0.882	0.927	0.855	0.904	0.884	0.930	0.849	0.906
Stapler	455	535	0.954	0.966	0.923	0.960	0.952	0.964	0.909	0.958
Stomach	2820	4356	0.884	0.887	0.815	0.885	0.886	0.889	0.783	0.887
Trocar	284	326	0.852	0.762	0.795	0.805	0.866	0.775	0.801	0.818

AP@[0.50–0.95]. Average precision at Intersection over Union (IoU) 0.50–0.95

At the class level, instruments exhibited the highest detection AP@[0.50–0.95]: ultrasonic scalpel 0.949, hook 0.947, stapler 0.923, needle holder 0.927, forceps 0.909, and aspirator 0.891 (Table 1). The needle remained challenging (0.644). Organ categories were comparatively lower: omentum 0.702, colon 0.763, liver 0.801, stomach 0.815, intestine 0.822, and pancreas 0.855 (Table 1). Segmentation followed the same ordering with modest absolute decrements (e.g., ultrasonic scalpel 0.923; stapler 0.909; forceps 0.847; liver 0.778; omentum 0.660; needle 0.499) (Table 1).

The quality of the category-level predictions is illustrated in the confusion matrix plot (Additional file 1**:** Fig. S3a), which demonstrates the model’s high classification accuracy, evidenced by the dominant diagonal trend. Major anatomical structures like the liver (*n*=6756) and stomach (*n*=4403) showed robust true positive rates, alongside frequently used tools such as forceps (n=6616). Misclassifications were predominantly attributable to confusion with the background, particularly for liver (*n*=2237), omentum (*n*=1278) and Hem-o-lok (*n*=1137), likely reflecting motion blur or partial visibility. Minor inter-class errors occurred between anatomically adjacent structures (e.g., pancreas-omentum, stomach-intestine). The normalized confusion matrix plot (Fig. 2**c**) showed that 17 of 18 categories were correctly classified with a normalized value of ≥0.85.

### Internal testing performance

**Fig. 3:**
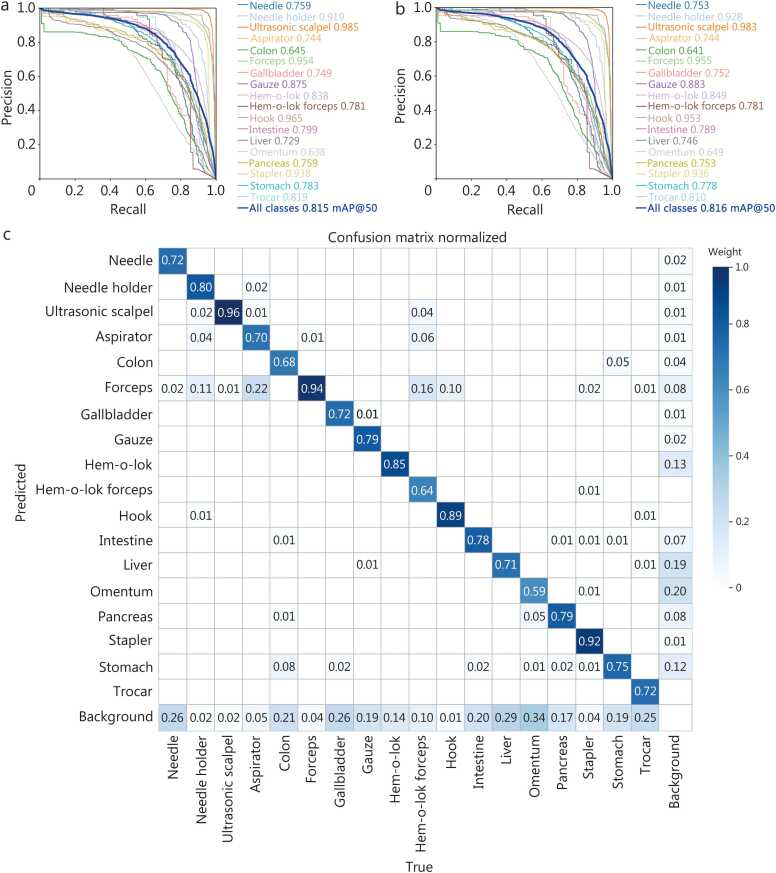
Internal testing set: precision-recall (PR) plot for box bounding (**a**) and mask segmentation (**b**), and normalized confusion matrix heatmaps (**c**). For the PR plot, the X-axis (“recall”) ranges from 0 to 1.0 and represents the proportion of actual positive instances (e.g., objects in the image) that the model correctly detects; higher recall indicates fewer missed objects. The Y-axis (“precision”) ranges from 0 to 1.0 and represents the proportion of the model’s positive predictions that are correct (i.e., low false-positive rates). The PR plots show the trade-off between precision and recall across different detection thresholds. Each subplot displays 18 distinct curves, each rendered in a different color (e.g., blue, orange, green, red, purple), corresponding to the 18 object classes. At the bottom of the legend, the overall mean average precision (mAP) for all classes is reported. For the normalized confusion matrix heatmaps, rows represent the predicted labels and columns represent the true (ground-truth) labels. Each cell’s color and value indicate the proportion of true instances that were predicted as a particular class. Values range from 0 to 1.0 and sum to 1.0 within each column. Cells are shaded along a blue gradient-light blue for low values (approximately 0) and dark blue for high values (approximately 1.0). Text in each cell displays the exact normalized value. Diagonal cells represent correctly classified instances (e.g., a diagonal value of 0.96 means that 96% of ultrasonic scalpel instances were correctly predicted). Off-diagonal cells represent misclassifications. For example, the cell at row “Forceps” and column “Aspirator” has a value of 0.22, indicating that 22% of all true aspirator instances were incorrectly predicted as forceps.

On the internal testing set, overall box bounding detection performance was precision 0.809, recall 0.748, mAP@50 0.815 (Fig. 3**a**), AP@[0.50–0.95] 0.670, and F1 score 0.777. Semantic mask segmentation performance was precision 0.813, recall 0.745, mAP@50 0.816 (Fig. 3**b**), AP@[0.50–0.95] 0.642, and F1 score 0.778 (Table 2). Relative to the training set, AP@[0.50–0.95] decreased by 0.176 for detection and 0.167 for segmentation.

**Table 2:** Internal testing set.

Category	Images	Label count	Box bounding metrics				Mask segmentation metrics			
			Precision	Recall	AP@[0.50–0.95]	F1 score	Precision	Recall	AP@[0.50–0.95]	F1 score
All	1803	17,211	0.809	0.748	0.670	0.777	0.813	0.745	0.642	0.778
Needle	217	295	0.806	0.619	0.501	0.701	0.796	0.607	0.350	0.688
Needle holder	276	294	0.905	0.813	0.828	0.857	0.916	0.820	0.795	0.866
Ultrasonic scalpel	684	706	0.968	0.956	0.923	0.962	0.968	0.953	0.910	0.961
Aspirator	135	138	0.680	0.746	0.704	0.712	0.686	0.746	0.646	0.715
Colon	330	429	0.636	0.669	0.487	0.652	0.640	0.660	0.482	0.650
Forceps	1601	3098	0.895	0.924	0.841	0.909	0.898	0.924	0.789	0.911
Gallbladder	97	101	0.701	0.703	0.600	0.702	0.718	0.693	0.612	0.705
Gauze	535	733	0.923	0.764	0.707	0.836	0.926	0.763	0.723	0.837
Hem-o-lok	787	1603	0.760	0.800	0.597	0.779	0.770	0.802	0.493	0.786
Hem-o-lok forceps	55	69	0.921	0.623	0.683	0.743	0.923	0.623	0.657	0.743
Hook	247	250	0.916	0.928	0.870	0.922	0.913	0.924	0.860	0.918
Intestine	534	1047	0.775	0.741	0.624	0.758	0.781	0.740	0.597	0.760
Liver	1233	2605	0.749	0.625	0.549	0.681	0.771	0.636	0.554	0.696
Omentum	1344	2851	0.738	0.521	0.433	0.611	0.747	0.520	0.424	0.614
Pancreas	710	1008	0.682	0.744	0.592	0.712	0.684	0.736	0.589	0.709
Stapler	193	227	0.882	0.912	0.846	0.897	0.883	0.912	0.830	0.897
Stomach	1137	1649	0.749	0.712	0.585	0.730	0.749	0.703	0.568	0.725
Trocar	101	108	0.878	0.665	0.691	0.757	0.865	0.651	0.686	0.743

AP@[0.50–0.95]. Average precision at Intersection over Union (IoU) 0.50–0.95

Instrument classes remained strongest by detection AP@[0.50–0.95] (Table 2): ultrasonic scalpel 0.923, hook 0.870, stapler 0.846, needle holder 0.828, and forceps 0.841. Mid‑range classes included gauze 0.707, aspirator 0.704, Hem‑o‑lok forceps 0.683, and trocar 0.691; the Hem‑o‑lok itself was lower (0.597). Needle performance was 0.501. Organ categories were consistently weaker: intestine 0.624, liver 0.549, stomach 0.585, pancreas 0.592, colon 0.487, and omentum 0.433. Error patterns suggested recall constraints for several lower‑performing categories (e.g., omentum recall 0.521; needle 0.619), whereas top instruments maintained high precision and recall (e.g., ultrasonic scalpel precision/recall 0.968/0.956). Segmentation mirrored detection trends with modest absolute reductions (overall AP@[0.50–0.95] 0.642 vs. 0.670).

The normalized confusion matrix (Fig. 3**c**) showed correct predictions in 96% of ultrasonic scalpel instances, 94% of forceps, 92% of staplers, and 89% of hooks. Hem-o-lok forceps had the lowest instrument-level accuracy (64%), with misclassifications to forceps (16%), aspirator (6%), and ultrasonic scalpel (4%) (Fig. 3**c**). Additionally, 22% of aspirators, 11% of needle holders, and 10% of hooks were misclassified as forceps (Fig. 3**c**). For organs, pancreas had the highest correct prediction rate (79%), while omentum had the lowest (59%) (Fig. 3**c**). The confusion matrix plot for raw label count is illustrated in Additional file 1**:** Fig. S3b**.**

### External validation performance

**Fig. 4:**
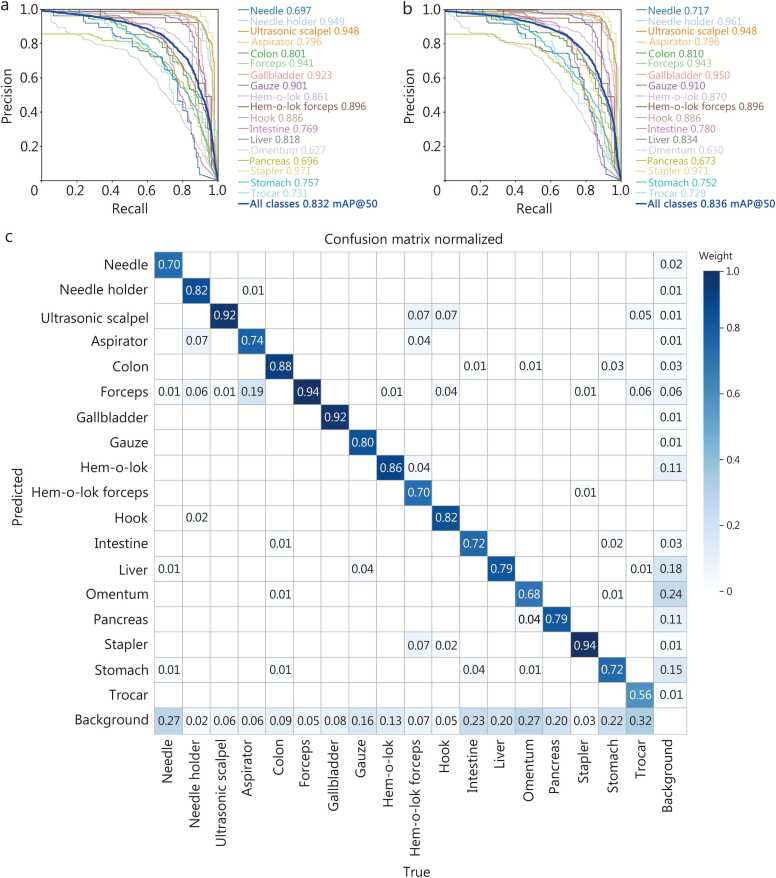
External validation set: precision-recall (PR) plot for box bounding (**a**) and mask segmentation (**b**), and normalized confusion matrix heatmaps (**c**). For the PR plot, the X-axis (“recall”) ranges from 0 to 1.0 and indicates the proportion of actual positive instances the model successfully detects. The Y-axis (“precision”) ranges from 0 to 1.0 and indicates the proportion of positive predictions that are correct. A higher value on either axis reflects better model performance. These PR curves illustrate how performance varies across detection thresholds. As in the previous figures, 18 distinct curves-each in a different color (e.g., blue, orange, green, red, purple)-represent the individual object classes. The overall mAP across all classes appears at the bottom of the legend. For the normalized confusion matrix heatmaps, rows represent the predicted labels and columns represent the true (ground-truth) labels. Each cell’s color and value indicate the proportion of true instances that were predicted as a particular class. Values range from 0 to 1.0 and sum to 1.0 within each column. Cells are shaded along a blue gradient-light blue for low values (approximately 0) and dark blue for high values (approximately 1.0). Text in each cell displays the exact normalized value. Diagonal cells represent correctly classified instances (e.g., a diagonal value of 0.94 means that 94% of stapler instances were correctly predicted). Off-diagonal cells represent misclassifications. For example, the cell at row “Forceps” and column “Needle holder” has a value of 0.06, indicating that 6% of all true needle holder instances were incorrectly predicted as forceps.

The external validation set comprised 721 images with 6685 label count (Table 3). Overall box bounding detection performance was precision 0.798, recall 0.773, mAP@50 0.832 (Fig. 4**a**), AP@[0.50–0.95] 0.684, and F1 score 0.785. Semantic mask segmentation performance was precision 0.806, recall 0.777, mAP@50 0.836 (Fig. 4**b**), AP@[0.50–0.95] 0.658, and F1 score 0.791. These results closely aligned with the internal testing set (detection AP@[0.50–0.95] 0.684 vs. 0.670; segmentation 0.658 vs. 0.642).

**Table 3:** External validation set.

Category	Images	Label count	Box bounding metrics				Mask segmentation metrics			
			Precision	Recall	AP@[0.50–0.95]	F1 score	Precision	Recall	AP@[0.50–0.95]	F1 score
All	721	6685	0.798	0.773	0.684	0.785	0.806	0.777	0.658	0.791
Needle	86	105	0.720	0.661	0.469	0.689	0.740	0.676	0.388	0.707
Needle holder	119	124	0.956	0.866	0.887	0.909	0.964	0.872	0.847	0.916
Ultrasonic scalpel	288	299	0.901	0.910	0.866	0.906	0.905	0.913	0.856	0.909
Aspirator	95	95	0.766	0.747	0.755	0.756	0.767	0.747	0.705	0.757
Colon	98	117	0.589	0.829	0.564	0.687	0.611	0.846	0.578	0.710
Forceps	603	1051	0.885	0.926	0.827	0.905	0.890	0.928	0.780	0.909
Gallbladder	128	134	0.840	0.888	0.719	0.863	0.863	0.910	0.778	0.886
Gauze	244	306	0.944	0.777	0.765	0.853	0.944	0.775	0.738	0.851
Hem-o-lok	363	726	0.783	0.818	0.709	0.800	0.792	0.825	0.595	0.808
Hem-o-lok forceps	24	27	0.917	0.814	0.785	0.863	0.916	0.812	0.731	0.860
Hook	55	55	0.839	0.836	0.827	0.838	0.841	0.836	0.852	0.839
Intestine	200	348	0.825	0.690	0.621	0.751	0.836	0.698	0.611	0.760
Liver	573	1043	0.767	0.735	0.635	0.751	0.787	0.750	0.610	0.768
Omentum	472	936	0.631	0.603	0.422	0.617	0.635	0.603	0.399	0.619
Pancreas	317	437	0.639	0.741	0.498	0.686	0.618	0.714	0.471	0.662
Stapler	67	71	0.854	0.972	0.870	0.909	0.855	0.972	0.877	0.909
Stomach	463	724	0.700	0.682	0.545	0.691	0.698	0.677	0.518	0.688
Trocar	81	87	0.812	0.425	0.548	0.559	0.844	0.437	0.490	0.578

AP@[0.50–0.95]. Average precision at Intersection over Union (IoU) 0.50–0.95

Instruments again led detection performance (Table 3): needle holder 0.887, stapler 0.870, ultrasonic scalpel 0.866, hook 0.827, and forceps 0.827. Mid‑range results were observed for gauze 0.765, aspirator 0.755, Hem‑o‑lok forceps 0.785, and Hem‑o‑lok 0.709. The needle remained challenging (0.469). Organ categories remained lower: liver 0.635, intestine 0.621, colon 0.564, stomach 0.545, pancreas 0.498, and omentum 0.422. Several classes were recall‑limited, notably trocar (recall 0.425). Segmentation followed detection with small absolute gaps overall; for instruments with clear boundaries, segmentation occasionally exceeded detection (e.g., hook 0.852 vs. 0.827; stapler 0.877 vs*.* 0.870), whereas organ classes showed larger segmentation decrements (e.g., omentum 0.399; pancreas 0.471).

The normalized confusion matrix (Fig. 4**c**) showed correct predictions in 94% of forceps, 94% of staplers, and 92% of ultrasonic scalpel, as well as 92% of gallbladders, 88% of colons, and 79% of pancreas. The corresponding raw label count confusion matrix can be found in Additional file 1**:** Fig. S3c**.**

### Real-time intraoperative deployment and clinical relevance

**Fig. 5:**
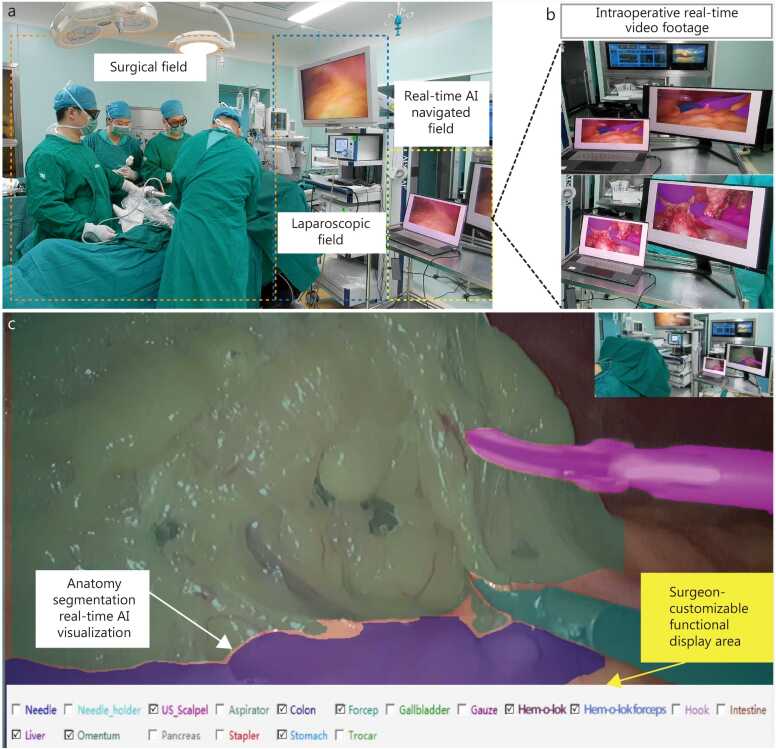
Intraoperative layout and real-time interface of the AI-assisted navigation system during totally laparoscopic distal gastrectomy. **a** Intraoperative layout: a working triangle centered on “Surgeon-Laparoscopy-AI system”, illustrating how the navigation interface integrates into the operative field. **b** Multimodal surgical field fusion display: combined presentation of conventional laparoscopy and AI-generated overlays. **c** Real-time interface: multimodal fusion display with the “On-Demand Display” feature, allowing selective visualization of specific organs or instruments during navigation. AI. Artificial intelligence.

We integrated the UI into the laparoscopic stack and performed laparoscopic gastrectomy with real-time AI assistance in a consecutive cohort of 15 patients [Fig. 5**;** Additional file 4 (https://figshare.com/s/4e8b769f33f8269c1f8a)]. The median postoperative hospital stay was 7 d (interquartile range: 6–9 d). There were two postoperative complications (surgical site infection and abdominal bleeding), both graded as Clavien-Dindo II and managed conservatively (Additional file 1**:** Table S1). The NASA-TLX, a validated multidimensional assessment of surgeon cognitive workload, yielded a mean overall workload score of 26.72±6.51 (scale: 0–100, with lower scores indicating lower perceived workload), indicating that the AI system did not substantially increase cognitive burden. The SUS score was 78.33±5.20, falling within the “good to excellent” usability range. No surgeon reported noticeable delay or that AI overlays were distracting. Infrequent misclassifications did not adversely affect decision-making because surgeons maintained independent visual assessment and used the AI system as supplementary confirmation. The average processing time was (31.8±10.7) ms per frame.

## Discussion

In this multicenter study, we developed, externally validated, and deployed an AI-based semantic segmentation system capable of recognizing 18 instrument and anatomic classes in real time during laparoscopic gastrectomy. On external validation, the model achieved an mAP@50 of 0.832 and a box-level F1 score of 0.785, comparing favorably with prior gastric surgery computer-vision tools [24]. The model demonstrated strong and stable recognition of surgical instruments, comparatively lower performance for organ structures and the needle, and closely matched internal and external results, supporting generalizability across datasets. This study demonstrates its feasibility and lays the groundwork for clinical translation.

Our external validation performance compares favorably with established systems in other surgical specialties. Tomita *et al.* [25] reported a mean IoU of 0.573 for vascular structure recognition in robotic pancreatoduodenectomy, whereas our system achieved an mAP of 0.836 at an IoU threshold of 0.50 with more complex multi-organ recognition across 18 categories. In colorectal surgery, recent AI-based systems achieved precision values of 0.707 for the ureter and 0.656 for the aortic plexus, though with fewer target classes [26]. Tokuyasu *et al*. [27] reported landmark detection performance during laparoscopic cholecystectomy (AP for the common bile duct: 0.320, AP for the liver: 0.314), demonstrating potential to reduce bile duct injury incidence and enhance surgical safety. Notably, our system’s distinguishing strength lies in maintaining robust detection and segmentation performance across 18 distinct classes while operating at real-time speeds (31.8 ms/frame), which is comparable to autonomous surgery systems requiring 40 ms/frame inference [28].

The approach delivers high pixel-level accuracy across essential anatomy and commonly used instruments, addressing the breadth of visual needs during gastric resection. As the number and diversity of classes increase, detection typically becomes more challenging, and few systems have attempted more than 10 classes. Using bounding box detection, Yamazaki *et al.* [29] reported a YOLOv3-based instrument detector with an mAP of 0.837 on test data. Restricting analysis to instruments, the external validation box mAP@50 in this multicenter study was 0.870, and the instrument-level mask mAP@50 reached 0.875. Semantic segmentation, by delineating object contours and spatial relationships at the pixel level, is inherently better suited for intraoperative navigation than bounding boxes alone [29]. Detailed pixel-level information will be essential for skill assessment, surgical autonomy, and instrument tracking. For organs, the mask mAP@50 (0.776) exceeded previously reported single-organ precision for pancreas (0.700) [24], [30]. Precise delineation of organ boundaries supports intraoperative decision-making, helping junior surgeons avoid misrecognition and alleviating cognitive load for expert surgeons.

Beyond mAP@50, we report AP@[0.50–0.95], box and mask F1 scores, and class-normalized confusion matrices, offering a multidimensional performance view and facilitating targeted model refinement. AP@[0.50–0.95], the mean AP across stringent IoU thresholds, is a more demanding and more representative metric than single-threshold AP@50 and is now standard in modern detection research [31]. In this study, AP@[0.50–0.95] values on external validation were in the high-0.6 range, aligning with state-of-the-art reports in complex surgical scenes [31], [32]. Several factors likely contributed to this robust cross-center performance. Chief among them is the quality and consistency of our ground-truth annotations. Each frame was segmented at the pixel level by board-certified gastrointestinal surgeons, minimizing label noise that has been shown to disproportionately degrade segmentation performance [33]. Such rigorous, surgeon-performed annotation is, however, difficult to scale. Expert annotation is resource-intensive [34], [35], and gastrectomy-specific datasets remain particularly scarce [23]. In contrast, AI research in laparoscopic cholecystectomy currently leads the field, largely because of the availability of publicly annotated datasets [36]. The shortage of diverse, high-quality annotated images remains a major challenge for developing and validating robust AI-based models in the surgical domain. To help address this gap, we provide our annotated frames publicly as supplementary material, which may be of high value for future algorithm development and computer-vision work in laparoscopic surgery.

AI-based systems may reduce intraoperative errors and improve safety through multiple mechanisms. Surgeon technical skills (dissection in the correct tissue plane) and non-technical skills (situational awareness and cognitive load management) are core elements of surgical performance [37]. AI-based visual guidance systems can assist in identifying anatomical structures and delineating surgical planes, thereby reducing intraoperative blood loss and minimizing organ injury [38]

**Fig. 6:**
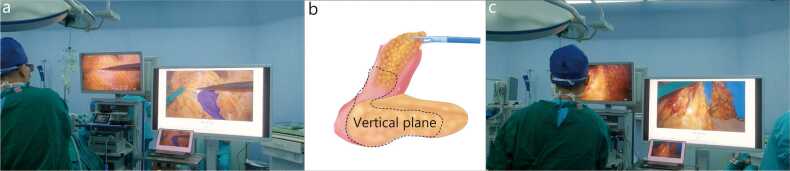
Visual guidance using the AI-based on-demand display. **a** AI-guided mobilization of the splenic flexure. Because of prior gastric perforation and dense adhesions, the colon boundary could not be reliably confirmed on the conventional display (left). The AI system (right) highlighted the colon boundary in soft violet, facilitating safe division of the gastrocolic ligament and exposure of the splenic vessels. **b** Maintenance of a vertical plane between the pancreatic head and the stomach during infrapyloric lymph node dissection, an essential step for safe and effective oncologic clearance. **c** AI-guided dissection in the infrapyloric area. On the traditional display (left), surgical planes can be difficult to discern. The AI system highlights the pancreas in light gray and the stomach in blue, creating a clear vertical dissection plane. This guidance enables the surgeon to separate fused mesentery safely and efficiently and facilitates identification of the right gastroepiploic vein. AI. Artificial intelligence.

Unlike many prior works focused on offline analysis or static risk prediction [39], [40], [41], the present system was deployed for real-time use in the operating room. Achieving stable performance in the operating room is nontrivial due to camera motion, occlusion, smoke, splashes, and variable lighting [42]. The UI integrated seamlessly into the standard laparoscopic stack using a single laptop-grade GPU, without altering routine workflow, suggesting that specialized computing racks are not a prerequisite for clinical integration. The algorithm recognized and highlighted key anatomic structures, instruments, and consumables, while the surgeon selectively toggled overlays (“on-demand display”) to focus on critical landmarks and suppress nonessential information to minimize cognitive load. The practical value of this capability is illustrated by a representative case in which a patient underwent gastrectomy for gastric cancer following a previous gastric perforation. Owing to a tortuous colon and dense adhesions in the splenic area, complete exposure of the left gastroepiploic vessels was not achievable using standard visualization alone. Real-time AI navigation clearly delineated the colonic boundaries (which appeared indistinct on conventional monitors), enabling safe and efficient division of the gastrocolic ligament while avoiding thermal injury to the colon (Fig. 6**a**). During subsequent dissection of the infrapyloric lymph nodes, where maintaining a strictly vertical dissection plane is crucial (Fig. 6**b**), the pancreatic plane highlighted by the AI system guided the surgeon to separate the fused mesentery along the correct anatomical layer, preventing entry into an improper plane that could result in hemorrhage or pancreatic injury (Fig. 6**c**). Prior work in laparoscopic cholecystectomy and left hemicolectomy has similarly shown reduced time to identify vital structures with AI guidance [26], [43], and the present study extends these benefits to gastric cancer surgery. The importance of such real-time visual support is underscored by data showing that 97% of bile duct injuries result from visual cognitive illusions [44], illustrating how fragile intraoperative perception can be even among experienced surgeons. Real-time AI assistance can provide cognitive support [43] that is particularly relevant for surgeons in tertiary hospitals, who typically face high workloads and burnout linked to cognitive strain, and it can improve communication and situational awareness among operating-room staff, further contributing to error reduction [45], [46].

Looking beyond immediate decision support, the same perceptual capabilities underpin the longer-term goal of surgical autonomy. High-fidelity scene understanding is foundational for autonomous surgery [47], and vision remains the principal bottleneck to higher levels of autonomy (LoAs) [48]. The extent of autonomy is classified into 6 LoAs, ranging from no autonomy (LoA 0), robot assistance (LoA 1), task autonomy (LoA 2), conditional autonomy (LoA 3), high autonomy (LoA 4), and full autonomy (LoA 5). Recent vision-based systems have demonstrated task or conditionally autonomous cholecystectomy (LoA 4) *in vivo* and *in vitro* [28], [49], in which target-object segmentation forms part of the input-perception module that subsequently drives robot action generation and execution. Reported inference speeds of approximately 40 ms per frame [28] are comparable to those of the present UI, indicating potential for integration into more complex algorithms to advance autonomous surgery for gastric cancer in the future.

These same capabilities may also help junior surgeons shorten their learning curves. First, AI systems can facilitate understanding of complex gastrointestinal surgical anatomy [50]. Second, the publicly available annotated dataset of 6832 frames addresses a critical gap in surgical education infrastructure. As highlighted by recent systematic reviews, the scarcity of high-quality annotated surgical datasets substantially limits both AI development and educational applications [51]. By providing expert-annotated frames encompassing 18 anatomical and instrument classes specific to laparoscopic gastrectomy, we enable the development of automated coaching systems capable of providing objective feedback without requiring constant senior surgeon supervision. Beyond intraoperative navigation, the UI can augment video-based coaching, which is an effective means of surgical skill development [52] but its implementation is limited by the availability of experienced coaches. The UI can analyze postoperative video to assist junior surgeons in self-learning anatomical structures and improving educational efficiency, particularly in settings with insufficient training staff. Operationally, the system runs on Windows; users load a video via “video,” select desired categories for real-time display, and automatically save annotated outputs to “recorded_videos”, while per-frame inference time and usage duration for nine instrument categories are logged (“processing_time” and “category_usage_time”). These logs enable quantitative analysis of the time spent by different surgeons on specific instruments, supporting objective skill assessment, cross-surgeon comparison and targeted refinement of surgical technique to shorten the learning curve [53]. Together with the on-demand display, these capabilities are consistent with recent evidence that AI assistance can elevate novice performance closer to expert levels [54].

Although developed on a gastrectomy dataset, the system’s “on-demand display” of organs and instruments is readily applicable to most abdominal procedures that share common landmarks, including pancreas identification during hemicolectomy and colon or small-bowel visualization during colectomy and small-bowel resection. Successful clinical implementation nonetheless requires addressing challenges beyond technical performance. The edge-computing approach performs all inference locally on a laptop-grade GPU, eliminating transmission of protected health information and keeping patient data stored on the user’s computer in compliance with institutional data governance. The system has successfully integrated with multiple commercial laparoscopic platforms through their standard serial digital interface outputs, without requiring specialized equipment. With respect to liability when AI guidance diverges from surgeon judgment, we emphasize that the system functions solely as a decision-support tool, with all final clinical judgment and surgical actions remaining under the direct control and responsibility of the operating surgeon.

## Limitations and future directions

Despite the promising results, there are several limitations. First, the external validation set, while derived from three independent institutions, comprises only one video per hospital (totaling 721 frames with 6685 annotated instances). Although this limited sample size restricts the ability to fully characterize inter-hospital performance variance, we have subsequently deployed the AI-based navigation system across different institutions using different commercial laparoscopic platforms (Aesculap EinsteinVision and Karl Storz systems), demonstrating practical generalizability. We provide the AI-based UI publicly to encourage multi-institutional validation studies. Second, misclassification was primarily driven by two factors: morphologic similarity among the aspirator, ultrasonic scalpel, and forceps at long working distances, and dynamic deformation of soft-tissue boundaries (e.g., omentum, liver edge) under retraction. These issues may be mitigated by integrating temporal context, audio signatures from energy devices, or deformable-convolution layers. Because of the inherent complexity of the surgical scene (occlusion, stains, blur, and brightness variation), image-enhanced methods could be explored to reconstruct images with denoising, deblurring, and color correction [55]. Third, while senior surgeons can readily identify and annotate certain anatomical structures, AI systems can detect them with high precision as well, and the reverse holds true for more challenging ones. The harder it is for surgeons to spot these structures, such as small nerves and blood vessels obscured by fatty tissue, the greater the potential benefit of AI-driven image navigation. A key challenge ahead involves labeling targets that even senior surgeons struggle to identify and training AI to recognize them. Clinically established fluorescent dyes, such as indocyanine green [56], may facilitate the delineation of these anatomical structures, thereby enhancing their identifiability. Fourth, although these preliminary results demonstrate feasibility and acceptable usability, larger prospective studies are needed to quantify effects on efficiency, error reduction, and clinical outcomes. Future randomized controlled trials should evaluate the impact on operative time, intraoperative adverse events, and postoperative complications.

## Conclusions

We developed a real-time, multiclass AI segmentation model for laparoscopic gastrectomy, demonstrated strong internal and external performance, and successfully deployed it at the bedside through a user-friendly UI. Together with our publicly available annotated dataset, this technology may facilitate safer dissection through intraoperative navigation, accelerate algorithm development, shorten learning curves, standardize operative quality, and advance progress toward autonomous actions in gastric cancer surgery.

## Abbreviations

AI: Artificial intelligence
YOLOv10: You Only Look Once version 10
JPEG: Joint Photographic Experts Group
IoU: Intersection over Union
AP: Average precision
mAP: Mean average precision
UI: User interface
NASA-TLX: National Aeronautics and Space Administration Task Load Index
SUS: System Usability Scale

## Ethics approval and consent to participate

The study was approved by the Institutional Review Board of the Chinese PLA General Hospital (S2024-076-01).

## Funding

This work was supported by the National Natural Science Foundation of China (82103593), and the “Three Talents and One Team” program (4143524).

## Data Availability

The authors confirm that the data supporting the findings of this study are available within the article its supplementary materials. Additional data can be accessed through Figshare links (https://figshare.com/s/0c0210ede8a309f83182; https://figshare.com/s/2cafd49139b6e6e02b41; https://figshare.com/s/4e8b769f33f8269c1f8a).

## Appendix A Supplementary material

Supplementary material

Supplementary material **Additional file 2. Dataset of annotated images for laparoscopic gastrectomy (**https://figshare.com/s/0c0210ede8a309f83182**).**

Supplementary material **Additional file 3. Inference UI of AI model (**https://figshare.com/s/2cafd49139b6e6e02b41**).**

Supplementary material **Additional file 4. Video presentation of real-time AI-assisted totally laparoscopic distal gastrectomy (**https://figshare.com/s/4e8b769f33f8269c1f8a**).**
