# Supplementary material for: Real-time multicenter semantic segmentation for laparoscopic gastrectomy: development, external validation and operating room deployment of an AI navigation system
Source: Mil Med Res. 2026 Jun 3;13(1):100040. doi: 10.1016/j.mmr.2026.100040 (PMC13440269; doi:10.1016/j.mmr.2026.100040)
Supplement: Supplementary file 1 [file mmc1.pdf]

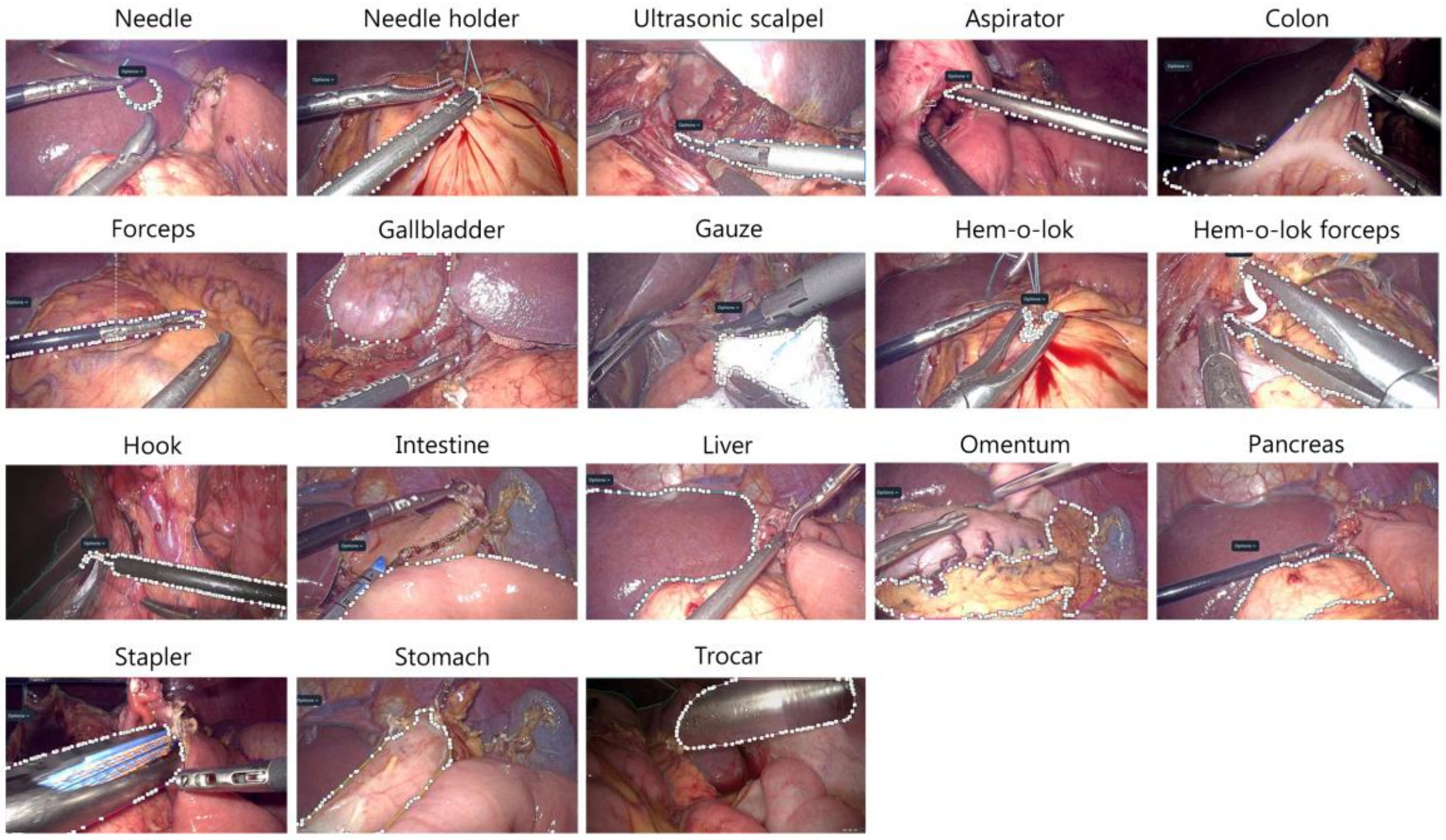

Fig. S1 Representative annotations for each category. The images depict real laparoscopic surgical scenes, with tools and organs annotated. All images originate from endoscopic camera views, characterized by dim lighting, reflections, blood, and fluids-factors that create realistic challenges for detection (e.g., occlusion, glare). The examples illustrate diversity in viewing angles and surgical states (e.g., instruments in active use vs. static anatomy)

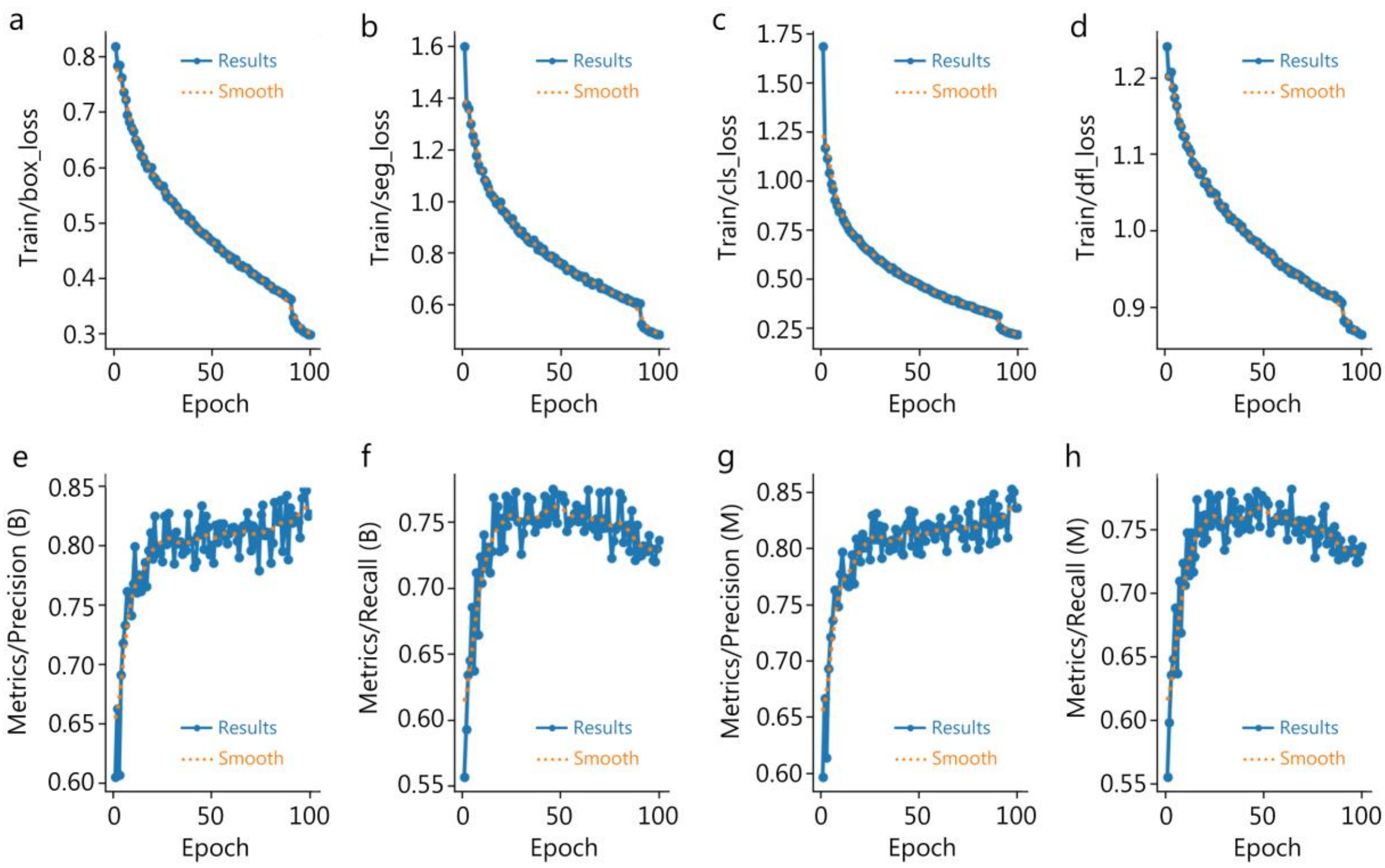

Fig. S2 Training summary plot. Training losses and performance metrics across epochs. **a** Train/box_loss: bounding-box regression loss, measuring how far predicted boxes deviate from ground truth (lower is better). **b** Train/seg_loss: segmentation (mask) loss, measuring pixel-wise agreement with ground-truth masks (lower is better). **c** Train/cls_loss: classification loss, reflecting how well the model predicts the correct class for each detection (lower is better). **d** Distribution focal loss (dfl): guides the predicted discrete distribution of box offsets toward the true bounding-box edges (lower is better). **e** Metrics/precision (B): box-level precision, fraction of predicted boxes that are correct detections at the chosen IoU threshold. **f** Metrics/recall (B): box-level recall, fraction of ground-truth boxes the model successfully detects. **g** Metrics/precision (M): mask-level precision, fraction of predicted masks that are correct detections. **h** Metrics/recall (M): mask-level recall, fraction of ground-truth masks successfully detected. Losses decrease smoothly across epochs, reflecting stable convergence without evidence of training instability

a

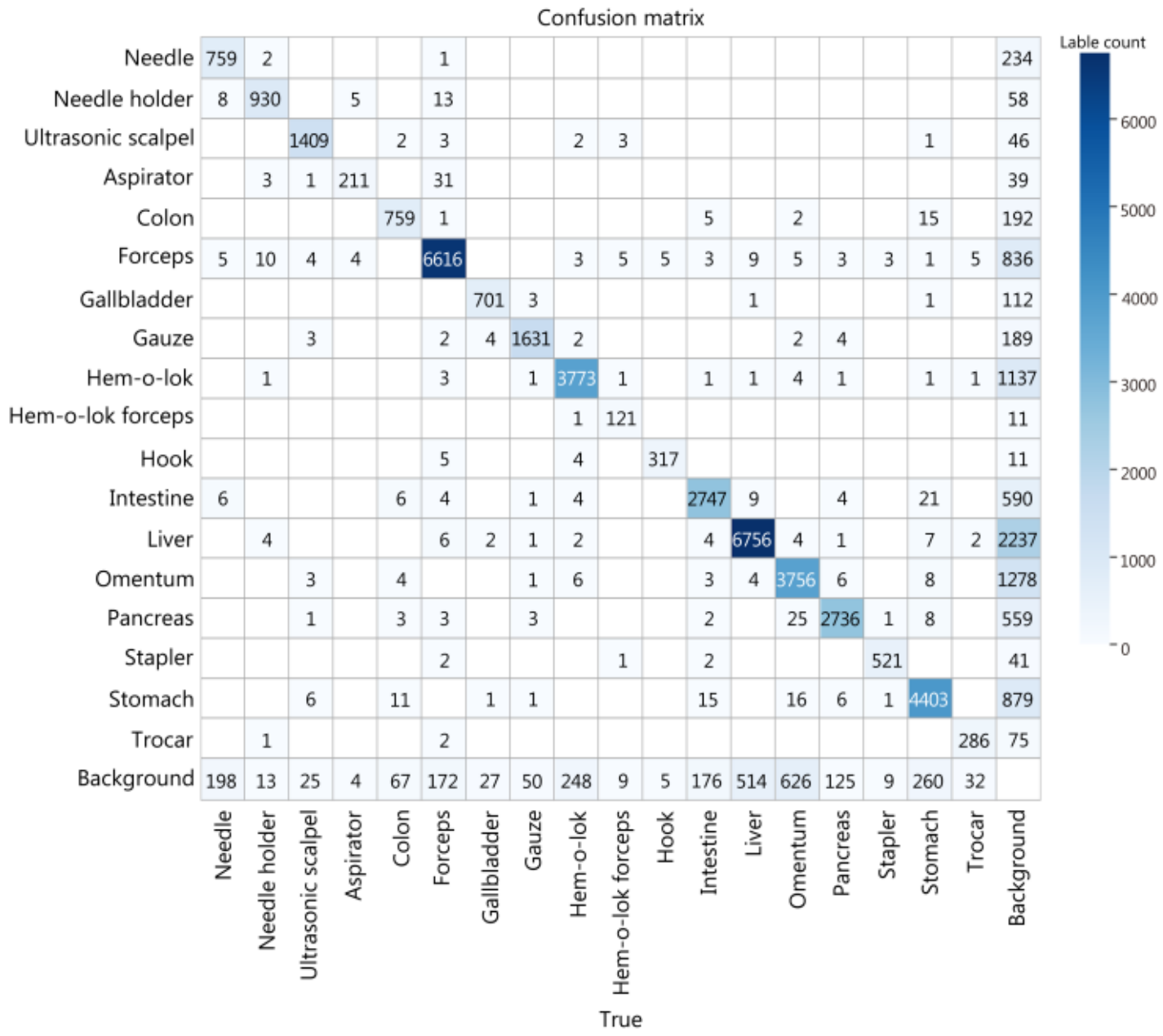
Confusion matrix
Lable count
Predicted
True
Needle
Needle holder
Ultrasonic scalpel
Aspirator
Colon
Forceps
Gallbladder
Gauze
Hem-o-lok
Hem-o-lok forceps
Hook
Intestine
Liver
Omentum
Pancreas
Stapler
Stomach
Trocar
Background

b

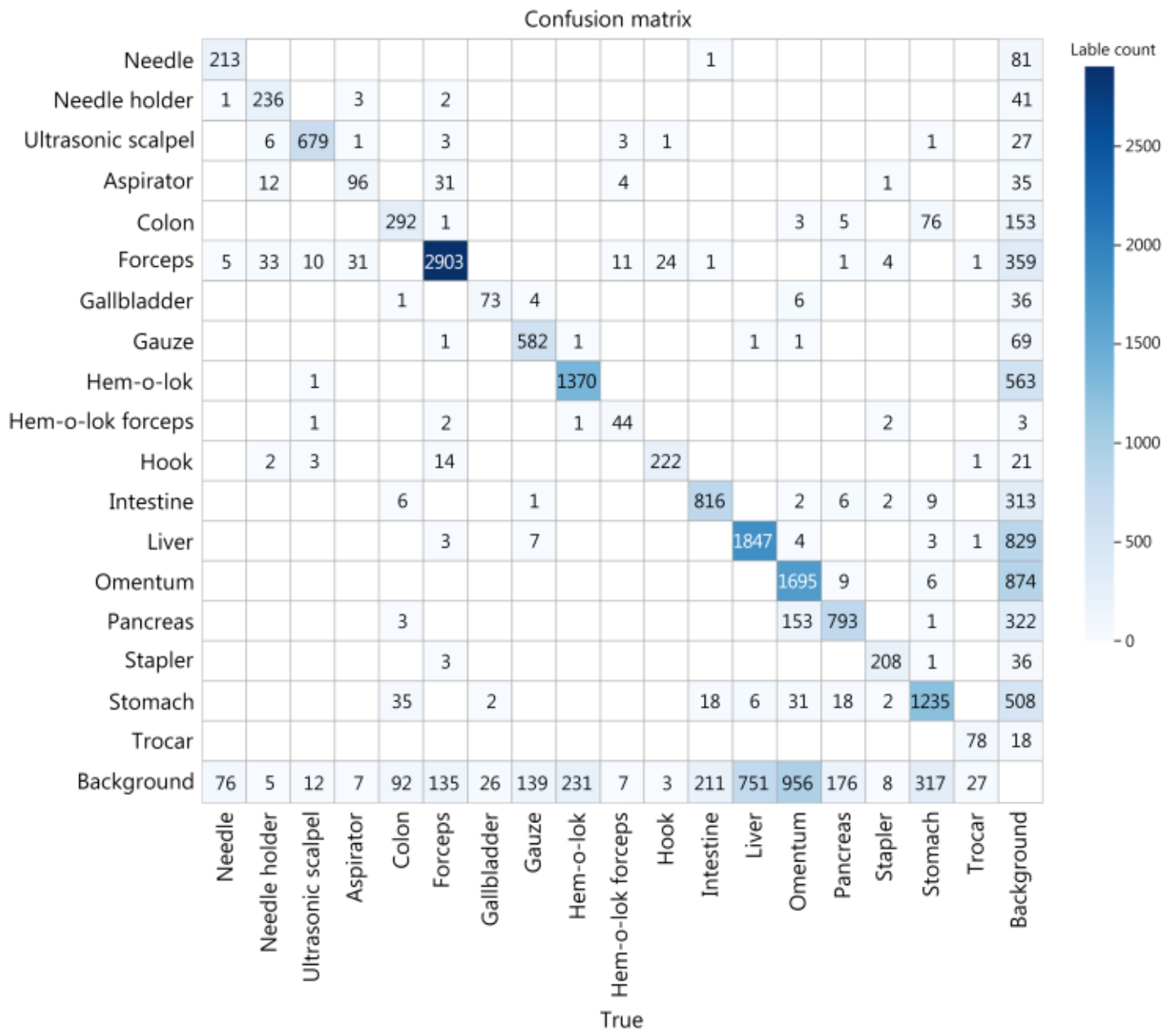
Confusion matrix
Lable count
Predicted
True
Needle
Needle holder
Ultrasonic scalpel
Aspirator
Colon
Forceps
Gallbladder
Gauze
Hem-o-lok
Hem-o-lok forceps
Hook
Intestine
Liver
Omentum
Pancreas
Stapler
Stomach
Trocar
Background

c

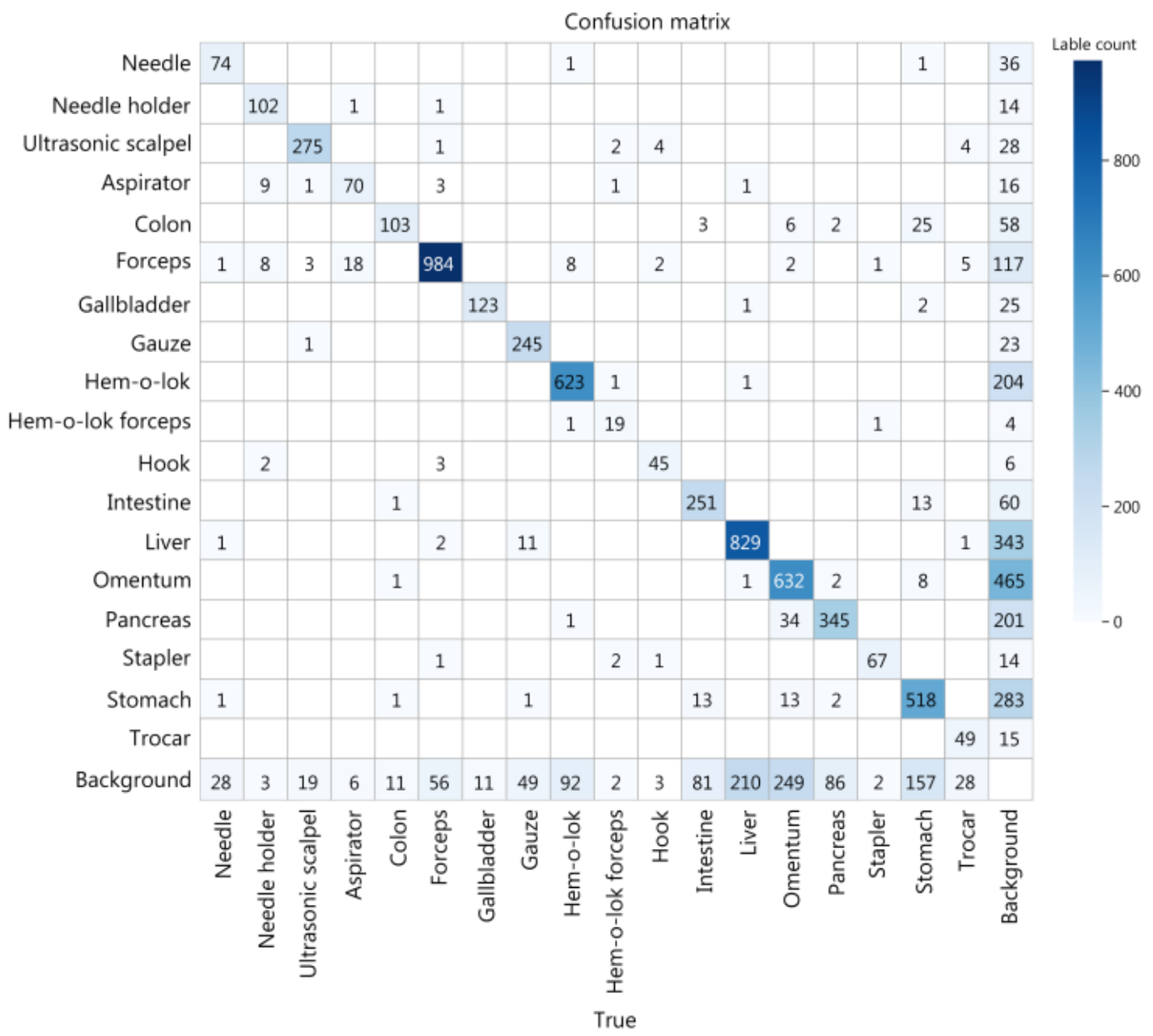
Confusion matrix
Lable count
Predicted
True
Needle
Needle holder
Ultrasonic scalpel
Aspirator
Colon
Forceps
Gallbladder
Gauze
Hem-o-lok
Hem-o-lok forceps
Hook
Intestine
Liver
Omentum
Pancreas
Stapler
Stomach
Trocar
Background

Fig. S3 Confusion matrix heatmap for the training set (**a**), internal testing set (**b**), and external validation set (**c**). Rows represent predicted labels and columns represent true (ground-truth) labels. Each cell shows the raw count of instances and is color-coded by magnitude. The matrices are diagonally dominant, indicating strong classification performance, with sparse off-diagonal entries reflecting low misclassification rates

Table S1 Clinic-pathological characteristic of patients underwent AI-assisted laparoscopic gastrectomy

Case No.	Operating time (min)	Intraoperative bleeding (ml)	Postoperative complications	Clavien-Dindo grading	Postoperative LOS (d)	Postoperative time to start clear liquid diet (d)	Time to removal of analgesic pump (d)	Time to first flatus (d)	(y)pT	(y)pN	(y) pTNM	Vascular invasion	Perineural invasion	Mental demand	Physical demand	Temporal demand	Performance	Effort	Frustration	Overall workload	System Usability Scale
1	115	50	No	-	6	1	3	3	1	0	I	No	No	12	11	9	9	14	13	11.33333	80
2	165	50	No	-	6	1	3	2	3	0	II	No	Yes	33	29	28	11	25	23	24.83333	75
3	130	100	No	-	6	2	3	3	2	0	I	No	Yes	25	31	26	13	23	27	24.16667	75
4	199	50	Surgical site infection	II	6	1	3	3	4	3	III	Yes	No	23	26	24	17	26	28	24	80
5	140	20	No	-	5	1	3	3	3	0	II	No	No	31	27	23	12	25	31	24.83333	72.5
6	208	300	No	-	9	4	3	3	3	3	III	Yes	Yes	47	44	34	18	36	41	36.66667	70
7	170	20	No	-	6	2	3	2	4	2	III	Yes	Yes	35	34	28	10	31	32	28.33333	72.5
8	132	50	No	-	6	1	3	3	4	3	III	Yes	Yes	18	22	18	11	19	21	18.16667	85
9	185	100	No	-	9	2	3	2	1	1	I	No	No	38	39	27	16	32	35	31.16667	90
10	155	20	No	-	7	2	3	3	1	0	I	No	No	21	28	18	14	29	28	23	77.5
11	154	50	No	-	8	1	3	2	4	0	II	No	Yes	34	35	22	15	36	28	28.33333	75
12	195	100	No	-	9	1	3	3	ypT0	0	ypT0N0	No	No	41	39	37	16	32	33	33	82.5
13	183	100	No	-	8	1	2	3	3	0	II	No	Yes	41	38	41	15	38	35	34.66667	80
14	169	50	No	-	9	1	3	2	4	1	III	Yes	Yes	37	36	38	8	38	31	31.33333	80
15	175	100	Bleeding	II	10	3	3	3	4	3	III	Yes	Yes	32	27	33	10	32	28	27	80

"-" represents blank. LOS. Length of stay

Questionnaire

NASA Task Load Index (NASA-TLX) + System Usability Scale (SUS)

Institution/Hospital: ____

Date: ____/____/_______

Name (optional): ____

Role: ☐ Attending ☐ Fellow ☐ Resident ☐ Other: ________________

Specialty/Service: ____

Procedure/Task Performed: _______________________________________

Case Type: ☐ Elective ☐ Urgent ☐ Emergency

PART A: Workload Assessment (NASA-TLX)

Instructions: Rate your experience for this specific case/task on each scale. Mark a number from **0 to 100**.

Dimension	Rating (0–100)
**1. Mental Demand**<br>How mentally demanding was the task?<br>(complexity, concentration, decision-making)	______/100
**2. Physical Demand**<br>How physically demanding was the task?<br>(posture, force, fatigue, fine motor effort)	______/100
**3. Temporal Demand**<br>How hurried or rushed was the pace?<br>(time pressure, urgency)	______/100
**4. Performance**<br>How successful were you at accomplishing the task?<br>(0=perfect, 100=failure)	______/100
**5. Effort**<br>How hard did you have to work to achieve your performance?	______/100

(mental + physical effort)

6. Frustration
How insecure, discouraged, irritated, or stressed did you feel? ______/100
(emotional/psychological stress)

Overall Workload Score (average of 6 ratings): ______/100

PART B: System Usability Scale (SUS)

Instructions: For each statement, circle the number that best represents your level of agreement.

1=Strongly Disagree | 2=Disagree | 3=Neutral | 4=Agree| 5=Strongly Agree

#	Statement	Rating
1	I think that I would like to use this system frequently.	1 2 3 4 5
2	I found the system unnecessarily complex.	1 2 3 4 5
3	I thought the system was easy to use.	1 2 3 4 5
4	I think that I would need the support of a technical person to be able to use this system.	1 2 3 4 5
5	I found the various functions in this system were well integrated.	1 2 3 4 5
6	I thought there was too much inconsistency in this system.	1 2 3 4 5
7	I would imagine that most people would learn to use this system very quickly.	1 2 3 4 5
8	I found the system very cumbersome to use.	1 2 3 4 5
9	I felt very confident using the system.	1 2 3 4 5
10	I needed to learn a lot of things before I could get going with this system.	1 2 3 4 5

SUS Score SUS: ______/100

PART C: Additional Feedback (Optional)

What did you like most about using this system?

What were the biggest challenges or frustrations?

What would you change or improve?

Would you recommend this system for clinical use?

☐ Yes ☐ No ☐ Unsure

Scoring Guide

NASA-TLX Score NASA-TLX:

- Add all 6 ratings and divide by 6
- Range: 0–100 (lower=better for workload)

SUS Score SUS:

1. For items 1, 3, 5, 7, 9: subtract 1 from rating
2. For items 2, 4, 6, 8, 10: subtract rating from 5
3. Sum all 10 converted scores (0–40 range)
4. Multiply by 2.5 to get final score (0–100)
