# Supplementary material for: Real-time multicenter semantic segmentation for laparoscopic gastrectomy: development, external validation and operating room deployment of an AI navigation system
Source: Mil Med Res. 2026 Jun 3;13(1):100040. doi: 10.1016/j.mmr.2026.100040 (PMC13440269; doi:10.1016/j.mmr.2026.100040)
Supplement: Supplementary file 2 [file mmc2.zip › mmc2/mmc2.docx]

<https://figshare.com/s/0c0210ede8a309f83182>
