# Supplementary material for: Real-time multicenter semantic segmentation for laparoscopic gastrectomy: development, external validation and operating room deployment of an AI navigation system
Source: Mil Med Res. 2026 Jun 3;13(1):100040. doi: 10.1016/j.mmr.2026.100040 (PMC13440269; doi:10.1016/j.mmr.2026.100040)
Supplement: Supplementary file 3 [file mmc3.zip › mmc3/mmc3.docx]

<https://figshare.com/s/2cafd49139b6e6e02b41>
